# The hepatoprotective activity of olive oil and *Nigella sativa* oil against CCl_4_ induced hepatotoxicity in male rats

**DOI:** 10.1186/s12906-016-1422-4

**Published:** 2016-11-04

**Authors:** Madeha N. Al-Seeni, Haddad A. El Rabey, Mazin A. Zamzami, Abeer M. Alnefayee

**Affiliations:** 1 Biochemistry Department, Faculty of Science, King Abdulaziz University, Jeddah, Saudi Arabia; 2 Bioinformatics Department, Genetic Engineering and Biotechnology Institute, Sadat City University, Sadat City, Minufiya Egypt

**Keywords:** Hepatoxicity, CCl_4_, Olive oil, *Nigella sativa*

## Abstract

**Background:**

Liver disease is the major cause of serious health problem leading to morbidity and mortality worldwide and the problem has increased in search for hepatotherapeutic agents from plants. The present study was designed to compare the probable hepatoprotective activity of olive oil and *N. sativa* oil on CCl_4_ induced liver damage in male rats.

**Methods:**

Forty males of a new model of albino rats (Wistar strain) (175–205 g) were divided into four groups. The 1st Group (G1) was the negative control group, the remaining rats were injected with CCl_4_ (1 ml/kg body weight) with equal amount of olive oil on the 1st and 4th day of every week for 4 weeks. The 2nd group (G2) was the positive control, the 3rd group (G3) and the fourth group (G4) were treated orally with *N. sativa* oil and olive oils using stomach tube.

**Results:**

The positive control group showed an increase in hepatic enzymes, total bilirubin, creatinine, uric acid, lipid peroxide total cholesterol, triglyceride, low density lipoprotein, very low density lipoproteins, interleukin-6, and a decrease in antioxidant enzymes, high density lipoprotein cholesterol, a decrease in total protein and albumin an when compared with negative control group. Histology of the CCl_4_ treated group revealed inflammation and damage of liver cells. Treating the hepatotoxic rats with olive oil and *N. sativa* oil showed a significant improvement in all biochemical tests compared with the positive CCl_4_ control group. In addition, the liver tissues of olive oil treated group showed mild improvement in inflammatory infiltration and in *N. sativa* oil treated group showed normal hepatocytes with no evidence of inflammation.

**Conclusion:**

This study revealed that olive oil and *N. sativa* oil have a protective effect against CCl_4_-induced hepatotoxicity in male rats. *Nigella sativa* oil was more effective than olive oil.

## Background

Liver is a multipurpose organ of the body that controls internal chemical environment [1]. It handles the metabolism and excretion of drugs and other xenobiotics from the body thereby providing protection against foreign substances by detoxifying and eliminating them [2]. Liver purifies enthetic chemical molecules through oxidation, reduction and/or conjugation [3]. It is certainly affected by free radical and causes disease hepatitis, cirrhosis, liver cancer and other alcohol related disorders [4]. Liver disease is the major causes of serious health problem leading to morbidity and mortality worldwide and the problem has increased in search for hepatotherapeutic agents from plants [5, 6].

Liver injury or dysfunction is well known as a serious health problem [7] and can be produced by toxic chemicals, drugs, and virus infiltration from ingestion or infection [8]. Exposure of diverse environment pollutants and xenobiotics such as alcohol, paracetamol, carbon tetrachloride (CCl4), thioacetamide are the major cause of liver disorder, which damage the liver by producing reactive oxygen species [5] which are extremely toxic and produce injury in the tissue through covalent bond and oxidation in DNA base, lipid and protein, Also can change the functional activity of enzymes and structural proteins [9]. Carbontetrachloride (CCl4) is hepatotoxicant and has been commonly used for generating liver injury in rat model [10]. In hepatocytes CCl4 is metabolized by the cytochrome P450 to produce the highly reactive free radicals [11]. The key role in the pathogenesis of CCl4-induced hepatic injury is oxidative stress, which is a physiological status associated with unbalance between free radical [10] and antioxidant defenses [12]. In addition, CCl4 hepatotoxicity also causes increased blood flow and cytokine accumulation that are characteristic of tissue inflammation.

For long time, plants were used in treatment of many diseases especially in the East region countries [13]. In addition, *Lycium barbarum* protects mice liver from carbon tetrachloride-induced oxidative stress and necroinflammation by reducing the hepatic necrosis and the serum alanine aminotransferase (ALT) level induced by CCl4 intoxication, inhibiting cytochrome P450 2E1 expression, and restoring the expression levels of antioxidant enzymes and decreasing the level of nitric oxide metabolism and lipid peroxidation induced by CCl4 [14].

Gorinstein et al. [15] stated that olive oils improve lipid metabolism and increase antioxidant potential in rats fed diets containing cholesterol. Administration of olive oil may have a potential role as an antioxidant and in lowering the risk of malignant neoplasms, especially breast and stomach cancer; and also in ovary, colon and endometrium cancer [16]. The popularity of olive oil is increasing mostly attributed to its antioxidant and anti-inflammatory effects, which may help in preventing diseases in humans [17]. Diverse studies have exposed that the consumption of olive oil may have a potential role in decreasing the risk of malignant neoplasms, especially breast and stomach cancer; and also in ovary, colon and endometrium cancer [18, 19].

*Nigella sativa* L. (is also known as black seed or black cumin) seeds have curative potential as described in the Old Testament and in Islamic culture [20]. Black seed oil is traditionally used for enhancing immunity and combating inflammatory and respiratory diseases, among many disorders [21]. Thymoquinone, present in *N. sativa* oil, has growth inhibitory effects against a variety of cancerous cells through the inhibition of DNA synthesis and the induction of cell cycle arrest [22, 23]. *Nigella sativa* anti-inflammatory potential accounts for the observed analgesic, antidiabetic, and antihistaminic effects, and ability to alleviate diabetes, respiratory diseases, rheumatoid arthritis, multiple sclerosis, and Parkinson’s disease [24, 25].

The aim of this study is to compare the protective and curative effects of olive oil and *Nigella sativa* oil on CCl4 induced liver damage in male rats.

## Methods

### Animal

Forty males of a new model of albino rats (Wistar strain) weighing about 175–205 g were obtained from King Fahd Medical Research Center (KFMRC), King Abdulaziz University, Jeddah, Saudi Arabia. All the animal experiments were carried out under protocols approved by the Institutional Animal House of the University of King Abdulaziz at Jeddah, Saudi Arabia. The plan of our study specifically was approved by our institutional ethics committee at King Abdulaziz University (KAU-1435). The rats were housed in standard laboratory conditions at a temperature of (25 ± 3 °C), relative humidity (50–55 %) and a 12 h light/dark cycle (five rats / cage) 2 weeks before the start of the experiment. Cages, bedding, and glass water bottles (equipped with stainless steel sipper tubes) were replaced twice per week. Stainless steel feed containers were changed once a week. All animals fed standard nutritionally balanced diet and drinking water *ad libitum*.

### Conventional animal basal diet

Standard nutritionally balanced diet consisted of the following ingredients; 20.0 % protein, 4.0 % fat, 5.0 % fiber, 1.0 % vitamin mix, 3.50 % mineral mix, 0.25 % choline chloride and 66.25 % corn starch. Its energy equals 2850 kcal/kg. The diet manufactured by a Grain Silos and Flour Mills, in Jeddah, KSA. Basal diet food was stored in a dry place out of direct sunlight.

Carbon Tetrachloride (CCl4) were obtained from Merck Ltd., Coimbatore, Tamilnadu (India).

The olive oil was an extra virgin oil, produced from a local olive oil factory in Jeddah, Saudi Arabia. The quality of the used olive oil was tested to be sure that it is true extra virgin olive oil spectrophotometrically by having a stronger absorbance. *Nigella sativa* oil were purchased from a local herbal medicine shop in Jeddah, Saudi Arabia.

### Design of the experiment

Forty rats were divided randomly into four groups, each consists of ten rats as follows:

1. Group 1)G1(: The first group is untreated control group and was administered with olive oil (intraperitoneally injected at 8.00 Am in the 1st and 4th day of every week until the last day of the experiment) which was used as vehicle, and fed normal basal diet and water for 4 weeks.
  CCl4 (1 ml/kg body weight) was administered to animals of all the remaining groups at 8.00 Am in the 1st and 4th day of every week until the last day of the experiment by intraperitoneal injection with equal amount of olive oil.
2. Group 2 (G2) was the positive CCl4 control group and received only CCl4 (1 ml/kg body weight): olive oil (1:1) at 8.00 Am in the 1st and 4th day of every week intraperitoneally injected for 4 weeks.
3. Group 3 (G3) was injected with CCl4 [(1 ml/kg body weight): olive oil (1:1)] at 8.00 Am in the 1st and 4th day of every week intraperitoneally and cotreated daily with olive oil (1 ml/kg bw, for 4 weeks) orally using a stomach tube, at 8.00 Am. This group represents the positive control treated with olive oil which is well known with it is hepatoprotective effect.
4. Group 4 (G4) was injected with CCl4 [(1 ml/kg body weight): olive oil (1:1)] intraperitoneally injected and cotreated daily with (1 ml/kg b w) of *Nigella sativa* oil for 4 weeks orally using a stomach tube, at 8.00 Am.

### Samples collection and organs weight

At the end of the experiment rats were anesthetized using diethyl ether then, blood samples were collected from the heart of rat under anesthesia with diethyl ether. Serum was separated by centrifugation at 7000 rpm for 15 min at 4 °C. After collection of blood, anaesthetized animals were scarified by cervical dislocation. The abdomen was opened and the organs (Heart, liver, kidney sand testes) were rapidly dissected out, weighed and kept in saline. Apiece of liver was washed in sterile saline and fixed in 10 % buffered formalin for histopathological studies. A piece of liver was kept at ice-cold temperature to prepare to prepare liver tissue homogenate for antioxidant enzymes, lipid peroxide and interleukin-6 estimation.

### Food intake and water consumption

Food intake per cage was recorded once per week.

### Weight gain (g), body weight gain ratio (BWG%) and food efficiency ratio (FER)

Body weight gain (g), body weight gain ratio (BWG%) and food efficiency ratio (FER) were calculated as follows:

$$ \mathrm{Weight}\ \mathrm{gain}\ \mathrm{of}\ \mathrm{rats} = \mathrm{Final}\ \mathrm{weight}\ \mathrm{of}\ \mathrm{rats}\ \left(\mathrm{g}\right) - \mathrm{Initial}\ \mathrm{weight}\ \mathrm{of}\ \mathrm{rats}\ \left(\mathrm{g}\right) $$

$$ \mathrm{B}\mathrm{W}\mathrm{G}\%=\mathrm{Final}\ \mathrm{weight}\ \mathrm{of}\ \mathrm{rats}\hbox{-} \mathrm{Initial}\ \mathrm{weight}\ \mathrm{of}\ \mathrm{rats}/\mathrm{Initial}\ \mathrm{weight}\ \mathrm{of}\ \mathrm{rats}\ \mathrm{X}\ 100 $$

$$ \mathrm{F}\mathrm{E}\mathrm{R} = \mathrm{Weight}\ \mathrm{of}\ \mathrm{rats}\ \left(\mathrm{g}\right)/\mathrm{food}\ \mathrm{intake}\ \left(\mathrm{g}\right) $$

### Liver tissue homogenate

A piece of the liver tissue was cut into small pieces and washed with phosphate-buffered saline and then grinded in a homogenization buffer consisting of 0.05 M Tris-HCl pH 7.9, 25 % glycerol, 0.1 mM EDTA, and 0.32 M (NH4)2SO4 and containing a protease inhibitor tablet from Roche (Germany). The lysates mix was homogenized on ice using a Polytron homogenizer. The mix was sonicated in an ice bath to prevent overheating for 15 s followed by 5 min centrifugation at 12,000 rpm and 4^∘^C. The supernatant was aliquoted and stored at −80^∘^C.

### Liver enzymes

Serum alanine aminotransferase (ALT) was estimated according to the method of Schumann and Klauke [26] using human kit (Germany), serum aspartate transaminase (AST) was estimated according to the method of Bergmeyer et al. [27], using Swemed diagnostics kit (India) and serum alkaline phosphatase (ALP) was estimated according to the method of Rick [28] using Human Kit (Germany). Estimation was done according to the instruction of the supplier.

### Total proteins

Total protein and albumin were measured using commercial kits according to the instruction of the supplier. Total protein was quantified according to the method of Cannon et al. [29] using a Total protein kit Sigma-Aldrich (USA). Serum Albumins were estimated according to the method of Lee [30] using Sigma-Aldrich (USA) according to the instruction of the supplier.

### Total bilirubin

Total bilirubin was estimated according to the method of Balistreri and Shaw [31] using Human Kit (Germany) according to the instruction of the supplier.

### Kidney functions

Kidney functions parameters; creatinine, uric acid, blood urea were measured using commercial kits according to the instruction of the manufacturer as follows: i- Serum urea and uric acid were estimated according to the methods of Fawcett and Scott [32], Fossati et al. [33], respectively using Human kit (Germany), ii- Serum creatinine was estimated according to the method of Tietz [34] using Moody International creatinine kit (UKAS, Germany).

### Assessment of lipid profile

Lipid profile was determined by assessing serum TG, cholesterol, VLDL, LDL and HDL levels using commercial kits, following manufacturer’s instructions. Serum total cholesterol (TC), serum high density lipoprotein (HDL) and serum triglyceride (TG) were estimated according to the method of Young [35] using Spinreact kit (Spain) according to the instruction of the supplier. The value of serum low density lipoprotein (LDL) and serum very low density lipoproteins (VLDL) was calculated according to the equation of Srivastava [36] as follows:

i- LDL = TC – (HDL + TG\5).
ii- VLDL = TC-(LDL+ HDL).

### Antioxidants and lipid peroxide

Antioxidant enzymes (catalase, glutathione-S-transferase), and lipid peroxide were assayed in the serum and liver tissue homogenate colorimetrically using Biodiagnostic kit (Egypt), according to the instruction of the manufacturer. The calculations of catalase activity, glutathione-S-transferase activity and lipid peroxide concentration were estimated by the suitable equation of the kit.

### Interleukin-6

The proinflammatory cytokines IL-6 was estimated in the serum and liver tissue homogenate according to the method of Hirano [37] using R&D Systems Inc (United States) kits according to the instruction of manufacturer.

### Histopathological investigations

A piece of liver was fixed in 10 % formalin, dehydrated in gradual ethanol (50–99 %), cleared in xylene, and embedded in paraffin. Sections were prepared and then stained with hematoxylin and eosin dye for microscopic investigation [38].

### Statistical analysis

Data were analyzed using SPSS program. T-test and the mean ± SD were calculated, and then the data were analyzed using one way analysis of variance (ANOVA, *p* < 0.05) using the protected least significant difference (LSD) test using SAS software.

## Results

### Food intake

Table 1 shows the effect of treating CCl_4_ induced hepatotoxicity in male rats with olive oil and *N. sativa* oil for 4 weeks on food intake. The mean values of food intake (FI) were not significantly changed in the 1st, 2nd and 4th week as a result of CCl_4_ induced liver hepatotoxicity. Whereas in the 3rd week, the mean values of FI in the olive oil treated group (G3) and the *N. sativa* treated group (G4) were lower than that of the negative control. The differences were significant at 1 % (*P* < 0.05).

**Table 1:** Effect of treating CCl_4_ induced hepatotoxicity in male rats with olive oil and *N. sativa* oil for 4 weeks on food intake

Food Intake g/day	Statistics	G1	G2	G3	G4
		N. Control	P. Control	Olive oil	*N. sativa* oil
1st week	Mean ± SE	14.50 ± 0.22 ^a^	14.50 ± 0.223 ^a^	14.66 ± 0.21 ^a^	14.50 ± 0.23 ^a^
	LSD 0.05 = 0.719				
	T-test	-	0.00^NS^	−0.41^NS^	0.01^NS^
2nd week	Mean ± SE	16.16 ± 0.16 ^a^	15.83 ± 0.16 ^a^	15.50 ± 0.22 ^a^	15.50 ± 0.22 ^a^
	LSD 0.05 = 0.730				
	T-test	-	1.58 ^NS^	1.58 ^NS^	1.58 ^NS^
3rd week	Mean ± SE	19.16 ± 0.54 ^a^	18.16 ± 0.40 ^ab^	17.00 ± 0.44^b^	17.16 ± 0.40 ^b^
	LSD 0.05 = 1.128				
	T-test	-	3.87^**^	2.90^*^	2.23^*^
4th week	Mean ± SE	20.01 ± 0.00 ^a^	20.00 ± 0.00 ^a^	20.02 ± 0.00 ^a^	20.03 ± 0.00 ^a^
	LSD 0.05 = 0.251				
	T-test	-	0.00 ^NS^	0.02^NS^	0.01^NS^

Data are represented as mean ± SE. T-test values ^*^: significant at *P* < 0.05,^**^: significant at *P* < 0.01. ANOVA analysis: within each row, means with different superscript (a, b, c or d) are significantly different at *P* < 0.05, whereas means superscripts with the same letters mean that there is no significant difference at *P*> 0.05. *LSD* least significant difference, *N.S* non significant

### Water consumption

Table 2 shows the effect of treating CCl_4_ induced hepatotoxicity in male rats with olive oil and *N. sativa* oil for 4 weeks on the weekly water consumption. In the positive control group, water consumption in the 1st and 2nd weeks was similar to that of the negative control, whereas in the 3rd and 4th weeks it was significantly higher than that of the negative control. In G3 and G4, water consumption in 1st and 2nd weeks was significantly (in the 1st week for G3 and G4 and the 2nd week for G4) or non significantly (in G3 in the second week) higher than that of the positive control group. While, in the 3rd and 4th weeks the mean values of water consumption was highly significant (in the 3rd week for G3 and G4) or non significant (in the 4th week) compared with the positive control group.

**Table 2:** Effect of treating CCl_4_ induced hepatotoxicity in male rats with olive oil and *N. sativa* oil for 4 weeks on the weekly water consumption

Water consumed ml/day	Statistics	G1	G2	G3	G4
		N. Control	P. Control	Olive oil	*N. sativa* oil
1st week	Mean ± SE	33.33 ± 1.05 ab	32.50 ± 1.11 b	36.33 ± 0.88 a	36.33 ± 0.88 a
	LSD 0.05 = 3.257				
	T-test	-	0.41 NS	−4.60***	−2.49*
2nd week	Mean ± SE	33.66 ± 0.88 b	32.50 ± 1.11 b	34.83 ± 0.90 b	37.16 ± 0.79 a
	LSD 0.05 = 2.308				
	T-test	-	1.40 NS	−1.68 NS	−3.97**
3rd week	Mean ± SE	27.50 ± 1.11 b	38.00 ± 0.96 a	28.00 ± 1.00 b	29.16 ± 1.53 b
	LSD 0.05 = 3.766				
	T-test	-	−11.38***	5.59***	6.30***
4th week	Mean ± SE	27.50 ± 1.11a	32.00 ± 1.00 ab	30.00 ± 1.29 b	29.00 ± 2.08 b
	LSD 0.05 = 3.235				
	T-test	-	−2.18*	1.22 NS	1.04 NS

Data are represented as mean ± SE. T-test values ^*^: significant at *P* < 0.05,^**^: significant at *P* < 0.01, ***: significant at *P* < 0.001. ANOVA analysis: within each row, means with different superscript (a, b, c or d) are significantly different at *P* < 0.05, whereas means superscripts with the same letters mean that there is no significant difference at *P*> 0.05. *LSD* least significant difference, *N.S* non significant

### Total body weight

Results in Table 3 show the effect of treating CCl_4_ induced hepatotoxicity in male rats with olive oil and *N. sativa* oil for 4 weeks on total body weight. In G2, (the rats with CCl_4_ induced hepatotoxicity, the positive control) the total body weight was significantly (*p* < 0.001) higher than that of the negative control in all weeks. In G3 the total body week in the first week was significantly (*p* < 0.001) higher than that of the positive control, whereas in the other weeks the increase in total body weight was non significant. In G4, the total body weight in the 1st and 4th weeks was significantly (*p* < 0.05) higher than that of the positive control, whereas in the 2nd and 3rd weeks the increase in total body weight was non significant compared with that of the positive control group.

**Table 3:** The effect of treating CCl_4_ induced hepatotoxicity in male rats with olive oil and *N. sativa* oil for 4 weeks on total body weight

Total body weight (g)	Statistics	G1	G2	G3	G4
		N. Control	P. Control	Olive oil	*N. sativa* oil
1st week	Mean ± SE	178.66 ± 1.08 ^d^	201.33 ± 0.88 ^a^	190.16 ± 2.10 ^c^	197.50 ± 1.11 ^b^
	LSD 0.05 = 3.504				
	T-test	-	−19.79^***^	4.85^***^	2.49^*^
2nd week	Mean ± SE	181.66 ± 1.30 ^c^	198.50 ± 0.95 ^ab^	196.00 ± 3.10 ^b^	200.83 ± 1.44 ^a^
	LSD 0.05 = 4.444				
	T-test	-	−11.82^***^	0.88 ^NS^	−1.71 ^NS^
3rd week	Mean ± SE	186.00 ± 1.29 ^c^	201.16 ± 0.74 ^ab^	199.50 ± 3.33 ^b^	204.16 ± 1.74 ^a^
	LSD 0.05 = 4.875				
	T-test	-	−10.48^***^	0.56 ^NS^	−1.56 ^NS^
4th week	Mean ± SE	189.00 ± 1.50 ^c^	203.83 ± 0.54 ^b^	202.66 ± 3.06 ^b^	208.50 ± 1.66 ^a^
	LSD 0.05 = 4.450				
	T-test	-	−8.34^***^	0.39 ^NS^	−2.49^*^

Data are represented as mean ± SE. T-test values ^*^: significant at *P* < 0.05, ***: significant at *P* < 0.001. ANOVA analysis: within each row, means with different superscript (a, b, c or d) are significantly different at *P* < 0.05, whereas means superscripts with the same letters mean that there is no significant difference at *P*> 0.05. *LSD* least significant difference, *N.S* non significant

### Weight of organs

Table 4 shows the effect of treating CCl_4_ induced hepatotoxicity in male rats with olive oil and *N. sativa* oil for 4 weeks on organ weight. There were no significant difference between the weight of heart, liver, right kidney, left kidney, right testis and left testis in the positive control group and the negative control group.

**Table 4:** The effect of treating CCl_4_ induced hepatotoxicity in male rats with olive oil and *N. sativa* oil for 4 weeks on organs weight

Organs weight g	Statistics	G1	G2	G3	G4
		N. Control	P. Control	Olive oil	*Nigella sativa* oil
Heart	Mean ± SE	0.433 ± 0.091 ^b^	0.450 ± 0.018 ^b^	0.650 ± 0.022 ^a^	0.566 ± 0.033 ^ab^
	LSD 0.05 = 0.164				
	T-test	-	−0.18 ^NS^	−6.92^***^	−5.53^***^
Liver	Mean ± SE	4.100 ± 0.821^ab^	3.550 ± 0.172 ^b^	4.600 ± 0.177 ^ab^	5.133 ± 0.049 ^a^
	LSD 0.05 = 1.277				
	T-test	-	0.73 ^NS^	−3.50^**^	−9.89^***^
Right kidney	Mean ± SE	0.483 ± 0.101 ^b^	0.633 ± 0.021 ^ab^	0.683 ± 0.040 ^a^	0.600 ± 0.000 ^ab^
	LSD 0.05 = 0.160				
	T-test	-	−1.62 ^NS^	−0.88 ^NS^	1.58 ^NS^
Left kidney	Mean ± SE	0.516 ± 0.104 ^b^	0.683 ± 0.016 ^ab^	0.700 ± 0.025 ^a^	0.633 ± 0.021 ^ab^
	LSD 0.05 = 0.169				
	T-test	-	−1.53 ^NS^	−0.54 ^NS^	1.46 ^NS^
Right testis	Mean ± SE	0.933 ± 0.194 ^a^	0.965 ± 0.020 ^a^	1.166 ± 0.033 ^a^	1.166 ± 0.033 ^a^
	LSD 0.05 = 0.317				
	T-test	-	−0.15 ^NS^	−5.38^***^	−7.79^***^
Left testis	Mean ± SE	0.966 ± 0.201 ^a^	0.956 ± 0.019 ^a^	1.2000 ± 0.025 ^a^	1.200 ± 0.025 ^a^
	LSD 0.05 = 0.316				
	T-test	-	0.04 ^NS^	−7.30^***^	−7.52^***^

Data are represented as mean ± SE. T-test values ^**^: significant at *P* < 0.01, ***: significant at *P* < 0.001. ANOVA analysis: within each row, means with different superscript (a, b, c or d) are significantly different at *P* < 0.05, whereas means superscripts with the same letters mean that there is no significant difference at *P* > 0.05. *LSD* least significant difference, *N.S* non significant

Treating the hepatotoxic rats with olive oil and *N. sativa* oil in G3 and G4, respectively significantly (*P* < 0.01) increased the weight of heart, liver, right testis and left testis when compared with the positive control group, whereas the weight of right and left kidneys were non significantly changed.

### Physiological evaluations (body weight gain, body weight ratio and food efficiency ratio)

Table 5 shows the effect of treating CCl_4_ induced hepatotoxicity in male rats with olive oil and *N. sativa* oil for 4 weeks on physiological evaluations (body weight, BWG %, BWG% and FER). BWG, BWG%, FER and FER% in G2 were very high significantly decreased as a result of liver damage compared with the negative control group (G1). Treating the hepatotoxicity in G3 and G4 with olive oil and *N. sativa* oil, respectively significantly (*P* < 0.001) increased these parameters compared with the positive control group.

**Table 5:** The effect of treating CCl_4_ induced hepatotoxicity in male rats with olive oil and *N. sativa* oil for 4 weeks on body weight, BWG %, BWG% and FER

Biological evaluation	Statistics	G1	G2	G3	G4
		N. Control	P. Control	Olive oil	*Nigella sativa* oil
BWG g /4 week	Mean ± SE	10.333 ± 1.282 ^a^	2.500 ± 1.231 ^d^	12.500 ± 1.765 ^a^	11.000 ± 0.730 ^a^
	LSD 0.05 = 3.678				
	T.test	-	4.832^***^	−5.175^***^	−7.059^***^
BWG %	Mean ± SE	5.788 ± 0.721 ^a^	1.254 ± 0.612 ^b^	6.565 ± 0.914 ^a^	5.564 ± 0.349 ^a^
	LSD 0.05 = 1.962				
	T.test	-	5.285^***^	−5.332^***^	−7.323^***^
FER g/day	Mean ± SE	0.020 ± 0.002 ^a^	0.005 ± 0.002 ^b^	0.026 ± 0.003 ^a^	0.023 ± 0.001 ^a^
	LSD 0.05 = 0.012				
	T.test	-	4.832^***^	−5.367^***^	−7.416^***^
FER %	Mean ± SE	2.087 ± 0.259 ^a^	0.518 ± 0.255 ^b^	2.660 ± 0.375 ^a^	2.341 ± 0.155 ^a^
	LSD 0.05 = 0.839				
	T.test	-	4.734^***^	−5.251^***^	−7.274^***^

Data are represented as mean ± SE. T-test values ***: significant at *P* < 0.001. ANOVA analysis: within each row, means with different superscript (a, b, c or d) are significantly different at *P* < 0.05, whereas means superscripts with the same letters mean that there is no significant difference at *P*> 0.05. *LSD* least significant difference

### Liver enzymes, total protein, albumin and total bilirubin

Table 6 shows the effect of treating CCl_4_ induced hepatotoxicity in male rats with olive oil and *N. sativa* oil for 4 weeks on liver enzymes, total protein, albumin and total bilirubin. CCl_4_ induced hepatotoxicity in rats of the positive control significantly (*P* < 0.001) increased the mean values of ALT, AST and ALP, whereas decreased the mean values of total protein, albumin and total bilirubin compared with that of the negative control group. Treating the CCl_4_ induced hepatotoxicity in rats with olive oil and *N. sativa* oil in G3 and G4, respectively significantly (*P* < 0.001) decreased the mean values of liver enzymes (ALT, AST and ALP) and increased total protein, albumin and total bilirubin compared with that of the negative control. Treating the CCl_4_ induced hepatotoxicity in rats with *N. sativa* in G4 was more efficient than treating with olive oil in G3.

**Table 6:** The effect of treating CCl4 induced hepatotoxicity in male rats with olive oil and *N. sativa* oil for 4 weeks on liver enzymes, total proteins, albumin and total bilirubin

Liver enzymes U/l	Statistics	G1	G2	G3	G4
		N. Control	P. Control	Olive oil	*Nigella sativa* oil
AIT	Mean ± SE	25.16 ± 1.35^d^	71.00 ± 1.41^a^	58.83 ± 0.47^b^	52.16 ± 1.13^c^
	LSD0.05 = 3.275				
	T-test	-	−21.00***	7.15***	39.46***
AST	Mean ± SE	26.50 ± 0.76^d^	81.16 ± 1.66^a^	65.50 ± 0.99^b^	50.50 ± 1.11^c^
	LSD0.05 = 2.501				
	T-test	-	−44.47***	10.95***	23.91***
ALP	Mean ± SE	156.66 ± 2.17^d^	286.16 ± 2.82^a^	228.50 ± 2.50^b^	185.50 ± 2.43^c^
	LSD 0.05 = 8.122				
	T-test	-	−37.51***	14.90***	19.55***
Total protein	Mean ± SE	7.65 ± 0.04^a^	4.81 ± 0.09^d^	5.66 ± 0.04^c^	6.80 ± 0.05^b^
	LSD 0.05 = 0.201				
	T-test	-	22.55***	−10.04***	−16.22***
Albumin	Mean ± SE	4.50 ± 0.05^a^	2.56 ± 0.09^d^	3.10 ± 0.04^c^	3.85 ± 0.04^b^
	LSD 0.05 = 0.203				
	T-test	-	15.72***	−4.78***	−16.19***
Total Bilirubin mg/dl	Mean ± SE	0.42 ± 0.00^d^	1.38 ± 0.03^a^	0.84 ± 0.00^b^	0.76 ± 0.04^c^
	LSD 0.05 = 0.077				
	T-test	-	−28.02***	15.58***	15.11***

Data are represented as mean ± SE. T-test values ***: significant at *P* < 0.001. ANOVA analysis: within each row, means with different superscript (a, b, c or d) are significantly different at *P* < 0.05, whereas means superscripts with the same letters mean that there is no significant difference at *P* > 0.05. *LSD* least significant difference

### Renal function

Table 7 shows the effect of treating CCl_4_ induced hepatotoxicity in male rats with olive oil and *N. sativa* oil for 4 weeks on urea, creatinine and uric acid. CCl_4_ induced hepatotoxicity in rats of the positive control significantly at (*P* < 0.001) increased the mean values of urea, creatinine and uric acid compared with that of the negative control. Treating the CCl_4_ induced hepatotoxicity in rats with olive oil and *N. sativa* oil in G3 and G4, respectively significantly (*P* < 0.001) decreased the mean values of urea, creatinine and uric acid, when compared to that of the negative control. Treating the CCl_4_ induced hepatotoxicity in rats with *N. sativa* in G4 was more efficient than treating with olive oil in G3.

**Table 7:** The effect of treating CCl_4_ induced hepatotoxicity in male rats with olive oil and *N. sativa* oil for 4 weeks renal functions

Parameters mg/dl	Statistics	G1	G2	G3	G4
		N. Control	P. Control	Olive oil	*Nigella sativa* oil
Urea	Mean ± SE	24.00 ± 0.57 ^d^	55.50 ± 1.25 ^a^	43.83 ± 1.13^b^	32.66 ± 1.33 ^c^
	LSD 0.05 = 3.250				
	T-test	-	−21.65^***^	8.43^***^	16.30^***^
Creatinine	Mean ± SE	0.53 ± 0.02 ^d^	1.70 ± 0.05 ^a^	1.15 ± 0.04 ^b^	0.79 ± 0.01 ^c^
	LSD 0.05 = 0.117				
	T-test	-	−16.34^***^	11.00^***^	15.72^***^
Uric acid	Mean ± SE	4.18 ± 0.07 ^d^	6.61 ± 0.09 ^a^	6.01 ± 0.08 ^b^	5.10 ± 0.07 ^c^
	LSD 0.05 = 0.278				
	T-test	-	−15.56^***^	8.21^***^	10.48^***^

Data are represented as mean ± SE. T-test values ***: significant at *P* < 0.001. ANOVA analysis: within each row, means with different superscript (a, b, c or d) are significantly different at *P* < 0.05, whereas means superscripts with the same letters mean that there is no significant difference at *P* > 0.05. *LSD* least significant difference

### Lipid profile

Table 8 shows the effect of treating CCl_4_ induced hepatotoxicity in male rats with olive oil and *N. sativa* oil for 4 weeks on serum lipid profile. The mean values of TC, TG, LDL-C and VLDL-C in the positive control were significantly (*P* < 0.001) higher than that of the negative control. In contrast, the mean values of HDL in the positive control were significantly (*P* < 0.001) lower than that of the negative control (30.00 ± 0.57 and 48.33 ± 0.42 mg/dl, respectively).

**Table 8:** The effect of treating CCl4 induced hepatotoxicity in male rats with olive oil and *N. sativa* oil for 4 weeks on serum lipid profile levels

C	Statistics	G1	G2	G3	G4
		N. Control	P. Control	Olive oil	*Nigella sativa* oil
TG mg/dl	Mean ± SE	115.00 ± 4.64^d^	244.83 ± 4.77^a^	218.33 ± 15.12^b^	181.33 ± 1.97^c^
	LSD 0.05 = 26.170				
	T-test	-	−15.21***	1.50^NS^	9.80***
TC mg %	Mean ± SE	161.00 ± 3.06^d^	278.00±3.63^a^	227.00±2.06^b^	189.00±3.26^c^
	LSD 0.05 = 8.802				
	T-test	-	-22.16^***^	15.23^***^	16.90^***^
HDL mg/dl	Mean±SE	48.33±0.42^a^	30.00±0.57^d^	40.83±0.47^b^	36.50±0.42^c^
	LSD 0.05=1.554				
	T-test	-	24.11***	−18.02***	−7.67***
LDL mg/dl	Mean ± SE	89.33 ± 3.33^d^	198.50 ± 4.13^a^	145.50 ± 2.12^b^	115.83 ± 3.41^c^
	LSD 0.05 = 9.502				
	T-test	-	−17.86***	15.38***	14.68***
VLDL mg/dl	Mean ± SE	23.73 ± 1.56^c^	48.96 ± 0.95^a^	43.66 ± 3.02^c^	36.26 ± 0.39^b^
	LSD 0.05 = 5.595				
	T-test	-	−11.67***	1.50 ***	9.80***

Data are represented as mean ± SE. T-test values *: significant at *P* < 0.05,**: significant at *P* < 0.01, ***: significant at *P* < 0.001. ANOVA analysis: within each row, means with different superscript (a, b, c or d) are significantly different at *P* < 0.05, whereas means superscripts with the same letters mean that there is no significant difference at *P* > 0.05. *LSD* least significant difference, *N.S.* non significant

Treating the CCl_4_ induced hepatotoxicity in male rats with olive oil in G3 very high significantly (*P* < 0.001) decreased the mean values of TC, TG, LDL-C and VLDL-C compared with that of the positive control. In addition, the mean values of HDL in G3 were very high significantly (*P* < 0.001) higher than that of the positive control.

Similarly, treating the CCl_4_ induced hepatotoxicity in male rats with *N. sativa* oil very high significantly (*P* < 0.001) decreased the mean values of TC, TG, LDL-C and VLDL-C compared with the negative control values. Moreover, the mean values of HDL in G4 were significantly (*P* < 0.001) higher than that of the positive control. It is worthy to mention that *N. sativa* succeeded in lowering TC, TG, LDL-C and VLDL-C in G4 than olive oil in G3, whereas olive oil succeeded in increasing the levels of HDL than *N. sativa.*

### Antioxidant enzymes and lipid peroxide

Table 9 shows the effect of treating CCl_4_ induced hepatotoxicity in male rats with olive oil and *N. sativa* oil for 4 weeks on serum and liver tissue homogenate antioxidant enzymes and lipid peroxide. CCl_4_ induced hepatotoxicity in male rats of the positive control group (G2) significantly (*P* < 0.001) lowered the mean values of catalase (CA), superoxide dismutase (SOD) and glutathione reductase (GSST) and increased lipid peroxide in serum and liver tissue homogenate as a result of liver damage compared with the negative control group.

**Table 9:** The effect of treating CCl4 induced hepatotoxicity in male rats with olive oil and *N. sativa* oil for 4 weeks on CAT, SOD and GSST in serum and liver tissue homogenate

Parameters	Statistics	G1	G2	G3	G4
		N. Control	P. Control	Olive oil	*N. sativa* oil
Serum Catalase (S.CAT) U/I	Mean ± SE	2.77 ± 0.01^a^	0.13 ± 0.00 ^c^0	1.34 ± 0.05^d^	2.08 ± 0.06^b^
	LSD 0.05 = 0.438				
	T-test	-	189.86***	−25.73***	−35.40***
Serum Superoxide dismutase (S.SOD) U/ml	Mean ± SE	626.11 ± 3.88^a^	215.31 ± 3.15^d^	427.01 ± 2.99^c^	544.50 ± 3.87^b^
	LSD 0.05 = 9.935				
	T-test	-	91.91***	−58.51***	−66.38***
Serum Glutathione reductase (S.GSST) U/ml	Mean ± SE	717.81 ± 2.87^a^	246.68 ± 2.96^d^	518.28 ± 2.60^c^	631.68 ± 3.19^b^
	LSD 0.05 = 9.663				
	T-test	-	99.95***	−65.26***	−88.89***
CAT U/g. Liver tissue	Mean ± SE				
	LSD 0.05 = 0.230	5.53 ± 0.13^a^	.18 ± 0.01 ^d^0	2.38 ± 0.03^c^	4.58 ± 0.09^b^
	T-test	-	42.97***	−64.97***	−44.43***
SOD U/g. Liver tissue	Mean ± SE	815.06 ± 3.27^a^	213.81 ± 2.40^d^	628.48 ± 2.09^c^	745.80 ± 2.89^b^
	LSD 0.05 = 8.575				
	T-test	-	166.80***	−103.43***	−122.00***
GSST U/g. Liver tissue	Mean ± SE	734.76 ± 84.41^a^	373.33 ± 58.54^d^	669.95 ± 14.58^c^	739.01 ± 3.77^b^
	LSD 0.05 = 10.833				
	T-test	-	2.53***	−6.69***	−6.46***
MDA nmol/ml	Mean ± SE	0.39 ± 0.01^d^	2.85 ± 0.01^a^	1.50 ± 0.03^b^	0.88 ± 0.02^c^
	LSD 0.05 = 0.0817				
	T-test	-	−90.44***	40.04***	49.20***
MDA nmol/ g. Liver tissue	Mean ± SE	2.59 ± 0.12^d^	19.78 ± 0.37^a^	10.55 ± 0.37^c^	14.71 ± 0.17^b^
	LSD 0.05 = 0.977				
	T-test	-	−45.43***	13.65***	10.84***

Data are represented as mean ± SE. T-test values ***: significant at *P* < 0.001. ANOVA analysis: within each row, means with different superscript (a, b, c or d) are significantly different at *P* < 0.05, whereas means superscripts with the same letters mean that there is no significant difference at *P* > 0.05. *LSD* least significant difference

Treating the CCl_4_ induced hepatotoxicity in male rats in G3 and G4 with olive oil and *N. sativa* oil, respectively significantly (*P* < 0.001) increased the catalase, superoxide dismutase and glutathione reductase and decreased lipid peroxide in the serum and kidney tissue homogenate compared with that of the positive control group. In addition, *N. sativa* was more efficient than olive oil in ameliorating the antioxidant enzymes under study in G4 and G3, respectively.

### Interleukin-6

Data in Table 10 shows the effect of treating CCl_4_ induced hepatotoxicity in male rats with olive oil and *N. sativa* oil for 4 weeks on serum and tissue interleukin-6 (S.IL-6 and TIL6, respectively). The mean values of interleukin-6 (S.IL-6) of both serum and liver tissue homogenate in the positive control group were significantly (*P* < 0.001) higher than that of the negative control group which was in serum 21.96 ± 0.83 and 5.70 ± 0.21U/g, respectively and in tissue were 85.45 ± 3.11 and 43.05 ± 1.34 pg/ml, respectively. Treating the CCl_4_ induced hepatotoxicity in G3 rats with olive oil significantly (*P* < 0.001) lowered the mean values of S.IL-6 and T.IL-6 than that of the positive controls. Also, treating CCl_4_ induced hepatotoxicity in G4 with *N. sativa* oil significantly (*P* < 0.001) decreased the mean values of S.IL-6 and T.IL-6 compared with that of the positive control. *N. sativa* oil was more efficient than olive oil in ameliorating the levels of IL-6 in G4 and G3, respectively in both serum and liver tissue homogenate.

**Table 10:** The effect of treating CCl4 induced hepatotoxicity in male rats with olive oil and *N. sativa* oil for 4 weeks on serum and tissue tinterleukin-6 (S.IL-6 and TIL6)

Parameters	Statistics	G1	G2	G3	G4
		N. Control	P. Control	Olive oil	*N. sativa* oil
Interleukin-6 (S.IL-6) pg/ml	Mean ± SE	5.70 ± 0.21^d^0	21.96 ± 0.83^a^	16.78 ± 0.44^b^	10.48 ± 0.57^c^
	LSD 0.05 = 1.691				
	T-test	-	−18.38***	5.39***	10.98***
T.IL6 pg/g.tissue	Mean ± SE	43.05 ± 1.34^d^	85.45 ± 3.11^a^	64.80 ± 1.07^b^	56.10 ± 0.76^c^
	LSD 0.05 = 5.463				
	T-test	-	−11.36***	6.08***	1.20***

Data are represented as mean ± SE. ***: significant at *P* < 0.001. ANOVA analysis: within each row, means with different superscript (a, b, c or d) are significantly different at *P* < 0.05, whereas means superscripts with the same letters mean that there is no significant difference at *P* > 0.05. *LSD* least significant difference

### Histopathological investigation of the liver

**Fig. 1:**
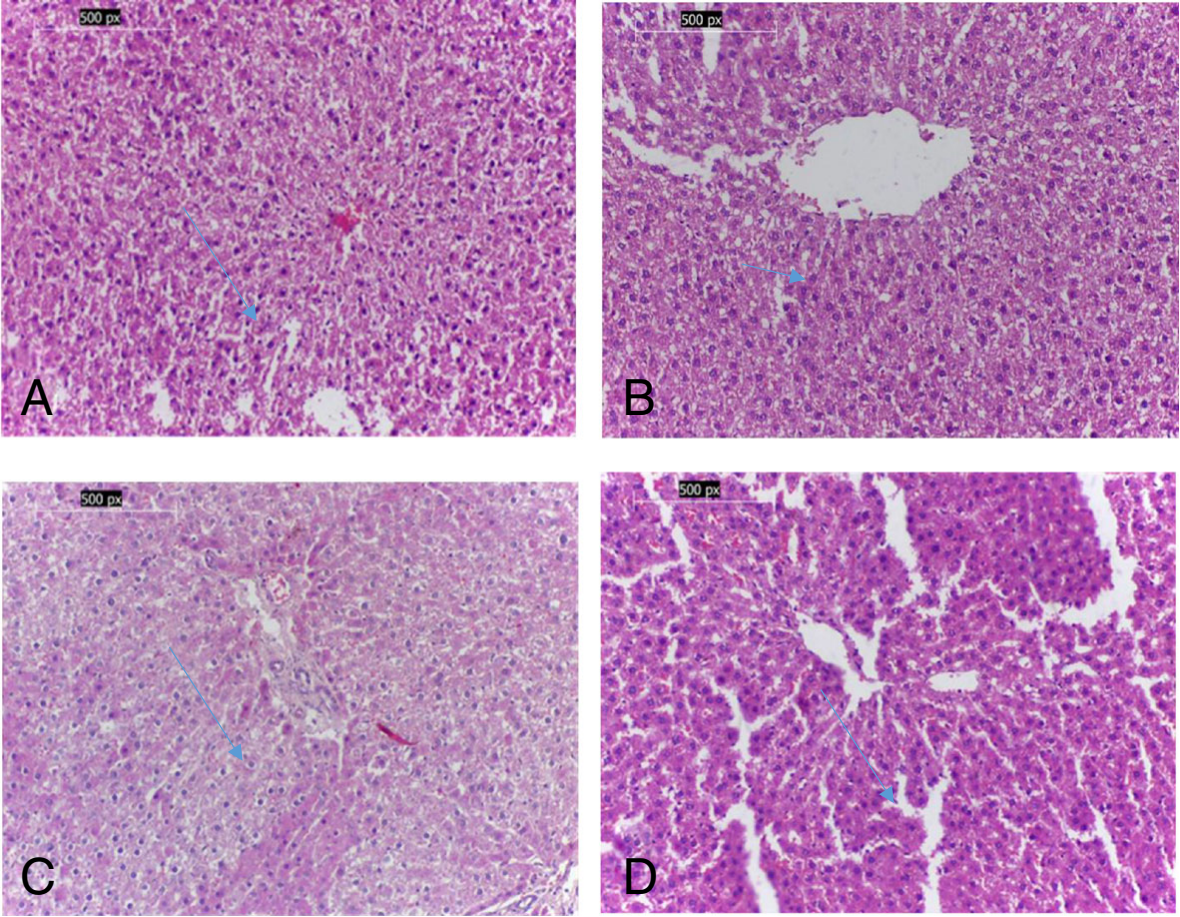
**a** Liver of the negative control (G1) showing no histopathological alteration with normal hepatocytes (*long arrow*), **b** liver of rat from the positive control group showing congested hepatocytes (*short arrow*) (G2), **c** liver of rat from (G3) group treated with olive oil showing nearly normal hepatocytes (*long arrow*), **d** liver of rat from (G4) group *Nigella sativa* showing normal hepatocytes (*long arrow*) (h & e, ×200)

Microscopically, liver of rats from G1 (negative control) showed normal architecture with portal tracts composed of normal bile duct, portal vein and hepatic artery. In addition, it showed normal kupffer cells (Fig. 1a). Whereas, photomicrography of the positive control representing CCl_4_ induced hepatotoxicity showed degeneration changes in hepatocytes with mild to moderate inflammation and congestion (Fig. 1b). In addition, disrupted hepatocytes and inflammatory cellular infiltration were also detected around the blood vessels as a result of induced hepatotoxicity. In Fig. 1c liver tissue of olive oil treated group showed improvement of the degeneration effect with minimal inflammatory infiltration. Figure 1d of *N. sativa* treated group showed nearly normal hepatocytes with no evidence of inflammation. This study indicates that olive oils and *Nigella sativa* has a protective effect against CCl_4_-induced impaired liver damage in male rats. *N. sativa* oil was more efficient than olive oil in ameliorating the liver tissues in G4 and G3, respectively.

## Discussion

In Saudi Arabia, the prevalence of liver diseases is relatively rising and the mortality and morbidity rates are significant [39, 40]. The present study was focused at studying the protective effect of olive oil and *Nigella sativa* oil to CCl_4_ induced hepatotoxicity in male rats. Liver injury can be induced directly from hepatic toxicity or indirectly from immune mediation by biological factors (e.g. hepatitis virus, bacteria, and parasite), environmental factors and chemical factors (e.g. medicine, industrial poisons and alcohol) [41].

In the current study, the positive control group rats given CCl_4_ showed a decrease in food intake. This result agrees with that of Wu et al. [42] and Tanaka et al. [43] who reported decreased food intake due to toxicity with CCl_4_. Moreover, decrease in food intake was detected after administration of olive oil and *Nigella sativa* to rats received CCl_4_. An increase in the total body weight in rats of the positive control group with induced CCl_4_ as compared with the negative control group was also encountered. Body weight loss is a distinctive feature of CCl_4_-induced hepatotoxicity [44]. One of the largest organ is liver that CCl_4_ administrated caused a rapid accumulation of triglycerides in the liver due to a block secretion of very low density lipoprotein by hepatocytes [45, 46]. Furthermore, increase in the total body weight was detected after administration of olive oil and *N. Sativa* oil to rats received CCl_4_.

In the current study, CCl_4_ induced liver damage in rats and consequently decreased BWG % which also accompanied with decreased FER compared with negative control group. The obtained results were in agreement with Fang et al. [17] and Khan et al. [47] who reported that CCl_4_ induced liver damage groups in rats showed significant reduction in body weight compared with rats non-injected with CCl_4_. Furthermore, increase in the body weight gain was detected after administration of olive oil. This result is consistent with that of Tufarelli et al. [48]. Furthermore, *N. Sativa* oil treated group (G4) showed very highly significant differences in BWG compared to the positive control group. This result is consistent with the study of Zaoui et al. [49]. Moreover, El-Sayed [50] discovered that the reduction seen in the body weight gain for 5 days after treatment with CCl_4_ that was alleviated by either *N. sativa* or thymoquione (one of the its major constituents) treatment on comparison with CCl_4_-treated animals. Our results showed that, no significant differences in heart, liver, kidneys and testes weight was observed after injection of CCl_4_ in rats for 28 daysays compared to the negative control. This agrees with the result of Kovalovich et al. [51].

The CCl_4_ injured liver functions showed significant increase in liver enzymes alanine aminotransferase (ALT), aspartate aminotransferase (AST) and alkaline phosphatase (ALP) compared with the negative control were observed after administration with CCl_4_ in the positive control as a result of hepatic cell damage. CCl_4_ induced hepatotoxicity and increased the aminotransferase and ALP activities and similar observation was found in group administrated with CCl_4_ and caused significant increase in ALT and AST enzymes in Wistar rats [52, 53]. Alanine aminotransferase is considered a highly sensitive and specific biomarker of hepatotoxicity. Elevation of ALP, a cell membrane enzyme is a primary marker of hepatobiliary effects and cholestasis [54]. Also, elevation in liver enzymes reflected liver cell damage and could be attributed to tissue breakdown, permitting the escape of intracellular enzymes from cytosol into the blood [52].

Treating the hepatotoxic rats in G3 and G4 with olive oil or *N. Sativa*, respectively reversed the activity of transaminases and restored them towards normal values indicating maintenance of functional integrity of hepatic cell membrane, however, they need a higher dose of *Nigella sativa* oil and olive oil to be restored to the normal levels. This agrees with our study which revealed that level of enzymes in CCl_4 +_ olive oil group (G3) and CCl_4 +_ *N. Sativa* oil group (G4) is lower than the CCl_4_ group (G2). These results agree with that of Krishnan and Muthukrishnan [55] who reported that AST, ALT and ALP elevated enzymatic levels were significantly returned toward normal levels by the 10 % aqueous extract of *N. sativa*.

On the other hand, renal function parameters in the present investigation showed a significant elevation in the level of uric acid (UA), urea and creatinine (CRE) when compared to that of the negative control. This indicates that the kidney was affected by CCl_4_ toxicity. UA and urea are the final product of nucleic acid or protein catabolism, respectively. The increased protein catabolism together with enhanced amino acid deamination for gluconeogenesis is possibly an acceptable postulate to understand the raised levels of urea. The elevated UA may be due to degradation of purines or to a rise of UA levels by either overproduction or inability of excretion. Moreover, 50 % of kidney function must be lost before an elevation in the serum CRE [56]. The current study shows that in spite of the ameliorative effect of both *Nigella sativa* oil and olive oil on kidney function parameters approaching the normal levels, they need a higher dose of *Nigella sativa* oil and olive oil to be restored to the normal levels.

The current findings indicated that there were a correlation between liver damage and kidney disease in this animal model that could be considered a novel study. On the other hand, the current results are consistent with other studies demonstrated a relationship between kidney disease and CCl_4_ liver toxicity [57–59]. Treating the damaged liver rats with olive oil and *N. sativa* oil protected the liver and improved the kidney function. Olive oil has been shown to reduce the kidney induced toxicity by a different nephrotoxin that resulted in reduced urea, CRE and UA levels [25, 60, 61]. These previous studies are consistent with our present study that showed reduced urea, CRE and UA levels in olive oil and *N. Sativa* oil group when compared with the positive control group in rats with CCl_4_ induced liver and injury and the administration of in olive oil and *N. Sativa* could recover the injury.

In the present study, serum concentration of total protein and albumin decreased after the injection of CCl_4_ in rats of the positive control group due to hepatotoxicity. The present result is in agreement with previous studies [52, 62]. CC1_4_ toxicity produced a significant decrease in plasma level of total protein and albumin. This may be as a result of releasing total protein and albumin from the cytoplasm into the blood quickly after cellular destruction and a reduction in forming hepatic protein [62]. Moreover, our results showed an increase in total protein and albumin after administration of olive oil and *N. sativa* oil to rats received CCl_4_. This result is also consistent with Al-Malki and El Rabey [25], Salem et al. [61] and Jin et al. [63].

In the current study, the CCl_4_ induced liver damage rats showed significant increase in total cholesterol (TC), triglyceride (TG), low density lipoprotein (LDL) and very low density lipoprotein (VLDL). In contrast, high density lipoprotein (HDL) was decreased compared with the negative control. This result is consistent with that of Hosseinzadeh et al. [64]. Treating these damaged-liver rats with olive oil and *N. sativa* oil significantly ameliorated the lipid profile parameters. The HDL was restored to the normal levels, whereas the other parameters need a higher dose of *Nigella sativa* oil and olive oil to be restored to the normal levels. This result is consistent with other studies [65, 66]. Oxidative stress due to CCl_4_ injection caused an increase in free fatty acid distribution to the liver and elevated hepatic TG accumulation and diet rich with olive oil and *N. Sativa* reduced the accumulation of TG in the liver [67].

In the current study, catalase (CAT), superoxide dismutase (SOD) and glutathione-S-transferase (GSST) were decreased in the serum and liver tissue homogenate of G2 as a result of CCl_4_ injection compared with negative control group, whereas lipid peroxide (MDA) in serum and tissue levels were increased. In spite of restoring the antioxidant enzymes to the normal levels, lipid peroxide needs a higher dose of *Nigella sativa* oil and olive oil to restore it to the normal levels. This result is consistent with that of Fang et al. [17]. Similar to the other above mentioned parameters, treating the CCl_4_ induced hepatotoxicity with olive oil or *N. Sativa* oil in G3 and G4, respectively significantly increased CAT, SOD and GSST and reduced MDA compared to the positive control group. This result is consistent with Krishnan and Muthukrishnan [55] and İlhan and Seçkin [67].

In the present study, CCl_4_-induced liver toxicity in male rats showed significant increase in both serum interleukin-6 (S.IL-6) and tissue interleukin-6 (T.IL-6) which is a proinflammatory cytokines compared with that of the negative control as a result of the inflammation occurred in the liver due to toxicity. This result is consistent with the increase in liver enzyme in the blood stream as a result of liver cells damage. Moreover, a decrease in the S.IL-6 and T.IL-6 levels was detected after administration of olive oil and *N. Sativa* to rats received CCl_4_. This agrees with other previous results [22, 23]. Several studies revealed the benefits of medical plants like olive oil or *Nigella sativa* (*N. Sativa*) oil on mice, rat and a rabbit model which showed anti-steatotic, anti-inflammatory and antioxidant effect and also delay in the development of liver disease [68–70].

Liver tissues showed many pathological changes as a result of CCl_4_ hepatotoxicity which is consistent with previous investigations [5, 8, 9]. Treating the CCl4 induced hepatotoxicity with olive oil or *N. Sativa* oil in G3 and G4, respectively significantly improved the liver tissues and nearly restored them to the normal. This result is consistent with other studies showed hepatoprotective role for both olive oil and *N. sativa* oil against pathological changes due to their higher content of antioxidant substance such as flavonoids and phenolic compounds [16, 17, 71]. To get full protection, the dose of *Nigella sativa* oil and olive oil may be increased to 1.5 ml /Kg body weight.

## Conclusion

This work showed that CCl_4_ causes liver hepatotoxicity as revealed by the elevation of liver function parameters and the adverse histopathogical changes in the liver tissues of rats of the positive control group. Treating the hepatotoxic rats with olive oil and *N. sativa* oil protected the liver structure against CCl4 toxicity in the third and the fourth groups, respectively. This hepatoprotective activity may be attributed to the biologically active compounds exist in both olive oil and *Nigella sativa* which work to scavenge free radicals. So, the current study recommends that the doses of olive oil and *N. sativa* oil should exceed the doses used in this study. The proposed dose is 1.5 ml/Kg body weight, in order to protect livers from the ecological hazards of CCl_4_ toxicity. It is also worthy to mention that olive oil is used here as the positive treatment due to its protective action to the liver. In addition, *N. sativa* is more efficient than olive oil in protecting the liver against CCl4 toxicity.

## Abbreviations

ALP: Serum alkaline phosphatase
ALT: Serum alanine aminotransferase
AST: Serum aspartate aminotransferase
B.W: Body weight
BWG: Body weight gain
BWG%: Body weight gain ratio (Percent)
Cat: Catalase
CCl_4_: Carbon tetrachloride
Crea: Creatine kinase-MB
CYP: Cytochrome P450
CYP-2E1: Cytochrome P450 2E1
FER: Food Efficiency ratio
FI: Food intake
G1: The first group, was untreated control group and was administered with (1 ml/kg body weight) olive oil (intraperitoneally injected)
G2: The positive CCl4 control group and received only CCl4 (1 ml/kg body weight): olive oil (1:1) on the 1st and 4th day of every week intraperitoneally injected for 4 weeks
G3: The 3rd group, was injected with CCl4 [(1 ml/kg body weight): olive oil (1:1)] intraperitoneally on the 1st and 4th day of every week and cotreated orally with olive oil (1 ml/kg bw) for 4 weeks using a stomach tube
G4: The 4th group, was injected with CCl4 [(1 ml/kg body weight): olive oil (1:1)] intraperitoneally injected and cotreated orally with (1 ml/kg b w) of *Nigella sativa* oil for 4 weeks using a stomach tube
GGT: Gamma-glutamyl transferase
GSH: Glutathione
GSST: Glutathione reductase
GSTs: Glutathione S-transferases
H&E: Hematoxylin and eosin
HDL: High density lipoprotein
LDL: Low density lipoprotein cholesterol
MDA: Malondialdehyde
*N. sativa*: *Nigella sativa*
ROS: Reactive oxygen species
S.IL-6: Serum interleukin-6
SOD: Superoxide dismutase
SPSS: Statistical program for sociology scientists
TC: Total cholesterol
TG: Triglyceride
TIL6: Tissue Tinterleukin-6
TQ: Thymoquinone
UA: Uric acid
VLDL: Very low density lipoproteins cholesterol
